# The association between age at menarche and depression: A systematic review and meta-analysis with meta-regression

**DOI:** 10.1016/j.clinsp.2025.100695

**Published:** 2025-05-23

**Authors:** Erfan Ghadirzadeh, Mahmood Moosazadeh, Kiarash Shakeriastani, Maryam Zarrinkamar, Mobina Gheibi, Forouzan Elyasi, Mojgan Geran

**Affiliations:** a Student Research Committee, Faculty of Medicine, Mazandaran University of Medical Sciences, Sari, Iran; b Psychosomatic Research Center, Sari Imam Khomeini Hospital, Faculty of Medicine, Mazandaran University of Medical Sciences, Sari, Iran; c Gastrointestinal Cancer Research Center, Non-Communicable Disease Institute, Mazandaran University of Medical Sciences, Sari, Iran; d Wake Forest Institute for Regenerative Medicine, Winston Salem, USA; e Department of Family Medicine, Diabetes Research Center, Mazandaran University of Medical Sciences, Sari, Iran; f Department of Medical Laboratory Sciences, Razi Hospital, Mazandaran University of Medical Sciences, Sari, Iran; g Department of Psychiatry, Psychiatry and Behavioral Sciences Research Center, Addiction Institute, Mazandaran University of Medical Sciences, Sari, Iran

**Keywords:** Depression, Menarche, Precocious puberty, Systematic review, Meta-analysis

## Abstract

**Introduction:**

The existing literature presents conflicting findings regarding the relationship between Age At Menarche (AAM) and depression. Thus, to address this gap, this systematic review and meta-analysis aimed to evaluate current evidence to clarify the association between AAM and depression.

**Methods:**

Medline (PubMed), Scopus, Embase, Web of Science, and Google Scholar were searched from 2000 until June 2024 to include cross-sectional, case-control, and cohort studies. The quality of the evidence was assessed using the Newcastle-Ottawa Scale (NOS) instrument. The odds ratio (OR) of depression and its 95 % Confidence Interval (95 % CI) were calculated using the random effects model and inverse variance method. The protocol is registered in PROSPERO, number CRD42024551838.

**Results:**

From a total of 2175 search records, 13 studies were included comprising 434,838 participants with NOS scores ranging from 7 to 9. The present findings showed that early menarche is associated with significantly higher odds of depression compared to both normative AAM (OR = 1.36, 95 % CI: 1.20‒1.53) and late AAM (OR = 1.52, 95 % CI 1.22‒1.90). Also, females with later menarche had lower odds of depression compared to females with normal AAM (OR = 0.91, 95 % CI 0.76‒1.09); however, this association was not statistically significant.

**Conclusion:**

The present findings demonstrated that early menarche is associated with elevated odds of depression compared to females of both normative AAM and late AAM.

## Abstract (author highlights)

• There is conflicting results regarding the relationship between AAM and depression.
• Thirteen observational studies were included comprising 434,838 participants.
• Findings showed that early menarche is associated with elevated odds of depression.
• Association between AAM and depression might present a dose-responsive behavior.

## Introduction

The timing of menarche is a critical marker of female pubertal development with substantial implications for both physical and mental health.^1^ The hormonal changes during puberty can lead to mood swings and increased emotional sensitivity, and adolescents may experience heightened levels of anxiety, sadness, or irritability.^2^^,^^3^ The physical changes during puberty can impact self-esteem and body image, which can affect their mental well-being.^4^

Research has indicated a link between the Age at Menarche (AAM) and the risk of psychiatric disorders.^5^^,^^6^ Puberty involves significant biological and psychological changes, including fluctuations in sex steroids such as estrogen, which have been implicated in the development of depressive symptoms.^7^, ^8^, ^9^ Variations in the timing of menarche, whether early or late, might therefore play a critical role in shaping mental health outcomes.

Primary studies suggest that both early and late menarche have been associated with an increased risk of depression and anxiety, potentially due to the social and biological stresses associated with off-timed pubertal development.^10^^,^^11^ From a clinical perspective, the identification of early menarche as a risk factor for depressive symptoms is particularly relevant. Early menarche, for example, could serve as a valuable alert for healthcare professionals to screen adolescents for depressive symptoms and provide timely interventions. Such proactive measures could help mitigate the long-term mental health risks associated with early pubertal development. However, the existing literature presents conflicting findings regarding the relationship between AAM and depression. Some studies suggest that early menarche increases the risk of depression, while others point to late menarche as a contributing factor.^12^^,^^13^ Additionally, several studies find no significant association between AAM and depression.^14^ Thus, to address this gap, this systematic review and meta-analysis aimed to evaluate current evidence to clarify the association between AAM and depression.

## Methods

This systematic review and meta-analysis of the relationship between AAM and depression was carried out following the Preferred Reporting Items for Systematic Reviews and Meta-Analyses (PRISMA) checklist to ensure transparent reporting.^15^ Also the review protocol has been registered on the PROSPERO database (CRD42024551838) for added transparency.

### Eligibility

The eligibility criteria are presented in Table 1.

**Table 1:** The eligibility criteria based on PECOS.

Population	**Exposure**	**Comparison**	**Outcome**	**Design**	**Language**
Individuals diagnosed with depression	Late AAM	Normative AAM	Number of participants with and without depression, stratified by early, late, and normative AAM	Case-control	English
	Early AAM			Cross-sectional	
				Cohort	

For the purpose of comprehensiveness of this study, no cut-offs for AAM were considered in the inclusion or exclusion of primary studies; however, in case a paper did not present data with specific cut-offs, the authors gathered data by the following definition: early menarche was defined as menarche occurring before the age of 12-years, normal menarche between 12- and 14-years, and late menarche as occurring after the age of 14-years.

### Search methods

To identify relevant studies, three independent reviewers searched major databases and search engines such as Medline (PubMed), Google Scholar, Scopus, Web of Science, and Embase from 2000 until 20 June 2024. Furthermore, the authors conducted a manual review of reference lists in pivotal articles and book chapters to ensure the inclusion of significant studies that may have been overlooked in electronic searches. To independently identify relevant literature, three investigators (MA, EG, and MG) employed specific keywords, their combinations, and related synonyms based on Medical Subject Headings (MeSH): age at menarche, menarche, menarche age, menarchal age, early menarche, late menarche, first menstrual cycle, menstruation, menstrual, depression, and depressive.

### Screening

Two reviewers (EG, and MG) initially screened the titles and abstracts of all retrieved records to identify relevant studies, checking for duplicates and irrelevance. They then independently assessed the full texts of promising articles to select the final studies. Any disputes were resolved by a third reviewer (FE). Meta-analyses, reviews, case reports, case series, animal studies, abstracts, conference papers, letters, editorials, and articles without accessible full texts were excluded.

### Data extraction

Three researchers (EG, KS, and MG) independently extracted information into a Microsoft Excel spreadsheet, including:

• Study characteristics (Author, country, publication year, population, and sample size);
• Study design (e.g., case-control, cohort, cross-sectional);
• Exposure (defined age ranges of AAM);
• Depression diagnosis tool;
• Outcomes (number of depressed and non-depressed patients by AAM type).

### Risk of bias

Two authors (EG and MZ) independently assessed potential bias in the included studies using the Newcastle-Ottawa Scale (NOS) checklists, which evaluated studies in three domains: selection, comparability, and outcome, with a maximum of nine points. The NOS checklist was adapted appropriately for different study designs (such as cross-sectional, cohort, or case-control studies). Studies scoring at least five points were included in the systematic review and meta-analysis. Disagreements were resolved by a third reviewer (MM) (Table 2).

**Table 2:** Results of the NOS quality assessment and detecting the Risk of Bias of the primary studies.

**Study**	**Cohorts**								**Final score**
	**Selection**				**Comparability**	**Outcome**			
	**Representativeness**	**Non-exposed selection**	**Exposure ascertainment**	**Outcome at baseline**	**Controlling for factors**	**Assessment**	**Follow-up duration**	**Lost to follow-up**	
Stice et al.^16^	*	*	*	‒	**	–	*	*	7
Herva et al.^17^	*	*	*	*	**	*	*	‒	8
Boden et al.^18^	*	*	*	*	**	*	*	‒	8
Opoliner et al.^19^	*	*	*	‒	**	*	*	‒	7
Sequeira et al.^20^	*	*	*	*	**	*	*	‒	7
	**Cross-sectionals**								
	**Selection**				**Comparability**	**Outcome**			
	**Representativeness**	**Size**	**Non-response rate**	**Ascertainment**	**Controlling confounders**	**Assessment**	**Statistics**		
Kaltiala-Heino et al.^21^	*	*	‒	**	*	**	*		8
Harlow et al.^22^	*	*	‒	**	*	**	*		8
Deng et al.^23^	*	*	‒	*	*	**	*		7
Jung et al.^24^	*	*	‒	**	*	**	*		8
Platt et al.^25^	*	*	*	**	*	**	*		9
Shen et al.^26^	*	*	*	**	*	**	*		9
Kim et al.^27^	*	*	‒	**	*	**	*		8
Umeda et al.^28^	*	*	‒	**	*	**	*		8

### Statistics

Utilizing Stata software version 11 (StataCorp, Texas, USA), the authors conducted data analysis. To determine the Odds Ratio (OR) of depression, a 2 × 2 table was utilized to extract the counts of positive and negative cases of depression in patients with early or late AAM, as well as in control group (normative AAM), from each primary study. The OR and its 95 % Confidence Interval (95 % CI) were calculated using the random effects model and inverse variance method. Heterogeneity in primary study results was assessed using the *I*-square and *Q* indices. Publication bias was examined through a funnel plot and Egger's test, and the Trim and Fill method was used to gauge its extent. Also, sensitivity analysis by leave-one-out method was performed to determine the impact of each primary study on the overall estimate. Subgroup analysis and meta-regression analysis were performed to evaluate the impact of population type (adults and adolescents), study design, and depression assessment tool (CES-D, DSM IV, and others) on results and to assess for potential sources of heterogeneity.

## Results

**Fig. 1:**
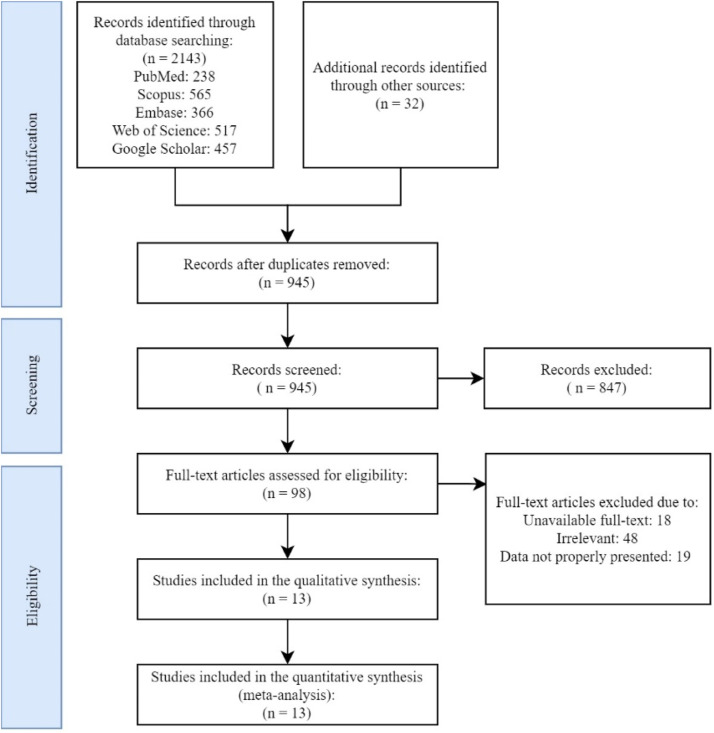
Search and selection flowchart of primary studies.

The authors identified 2175 records through this search. After screening titles and abstracts, the authors excluded 1230 duplicates and an additional 847 irrelevant records. Following a review of full-text articles for eligibility, 48 papers were excluded due to irrelevancy, 18 papers were excluded due to unavailability of their full-text, and 19 papers were excluded since they presented data in a way that was not extractable (for instance, only as figures, depression questionnaire scores, non-categorical presentation of AAM, etc.). Finally, thirteen studies satisfied the inclusion criteria consisting of five cohorts and eight cross-sectional studies with NOS scores ranging from 7 to 9 (Table 2 and Fig. 1). The primary studies were conducted from 2001 to 2022 in the USA (five papers), UK (one paper), Finland (two papers), New Zealand (one paper), China (one paper), Korea (two papers), and Japan (one paper). Seven studies were carried out on adolescent females aged below 18 years old, while six studies were conducted on adult females (Table 3).

**Table 3:** Characteristics of the primary studies.

**Study**	**Year**	**Country**	**Design**	**AAM definition**			**Population**	**Depression Diagnosis tool**	**Sample size**						
									**Total**	**Low AAM**		**Normal AAM**		**Late AAM**	
				**Early**	**Normative (control)**	**Late**				**D**	**Non-D**	**D**	**Non-D**	**D**	**Non-D**
Stice et al.^16^	2001	USA	CH	<11.6	≥11.6	ND	Girls aged 11‒15	K-SADS	496	35	130	37	294	ND	ND
Kaltiala-Heino et al.^21^	2003	Finland	CS	<12	12‒14	>14	Girls aged 14‒16	BDI	17,084	443	2940	1352	11,461	41	847
Harlow et al.^22^	2004	USA	CS	<12	12‒14	>14	Females aged 36‒45	CES-D	957	74	91	216	475	23	78
Herva et al.^17^	2004	Finland	CH	9‒11	12‒15	≥16	Females aged 31	HSCL-25	3952	90	392	544	2796	33	97
Boden et al.^18^	2011	New Zealand	CH	10‒11	12‒13	14‒15	Girls aged 15‒18	DSM-IV CIDI	497	29	46	94	211	38	79
Deng et al.^23^	2011	China	CS	<11	11‒14	>14	High school & College students	Zung SDS	8365	232	329	2167	4545	345	747
Opoliner et al.^19^	2014	USA	CH	≤12	12‒14.3	>14.3	Females aged 20‒25	CES-D	3711	105	486	408	2129	83	500
Jung et al.^24^	2015	Korea	CS	ND	≤14	≥15	Females aged 35‒74	CES-D	8472	ND	ND	126	2187	374	5785
Platt et al.^25^	2017	USA	CS	≤11	12‒13	≥14	Girls aged 13‒17	DSM-IV CIDI	4937	297	1085	473	2545	94	431
Sequeira et al.^20^	2017	UK	CH	ND	ND	ND	Girls aged 18	ICD-10 CIS-R	2208	44	310	136	1138	46	534
Shen et al.^26^	2019	USA	CS	<12	12‒13	≥14	Females aged ≥18	PHQ-9	15,674	455	2941	820	7010	455	4018
Kim et al.^27^	2021	Korea	CS	<12	≥12	ND	Girls aged 12‒18	WHO—CIDI	367,314	34,241	46,879	114,700	171,494	ND	ND
Umeda et al.^28^	2022	Japan	CS	≤10	>10	ND	Females aged 20‒75	DSM‒IV CIDI	1171	8	44	45	1074	ND	ND

D, Depressed; Non-D, Non-Depressed; CH, Cohort; CS, Cross-Sectional; AAM, Age At Menarche; ND, No Data; K-SADS, Adapted version of the Schedule for Affective Disorders and Schizophrenia for School-Age Children; BDI, Beck Depression Inventory; CES-D, Center for Epidemiological Studies for Depression; HSCL-25, Hopkins Symptom Checklist-25; DSM-IV, Diagnostic and Statistical Manual of Mental Disorders IV; CIDI, Composite International Diagnostic Interview; Zung SDS, Zung Self-Rating Depression Scale; ICD-10, 10th revision of the International Statistical Classification of Diseases and Related Health Problems; CIS-R, Clinical Interview Schedule-Revised; PHQ-9, Patient Health Questionnaire; WHO, World Health Organization.

### Early versus normal AAM

Twelve studies compared the odds of depression between early and normative AAM (seven studies in adolescence,^16^^,^^18^^,^^20^^,^^21^^,^^23^^,^^25^^,^^27^ and five studies in adults[17,19,22,26,28]). These studies included a total of 91,726 participants in the early AAM group and 326,164 individuals in the normal AAM group. In all of these twelve studies, the odds of depression were significantly higher among females with early AAM compared to those with a normal AAM; however, the results were statistically significant only in eight studies.

**Fig. 2:**
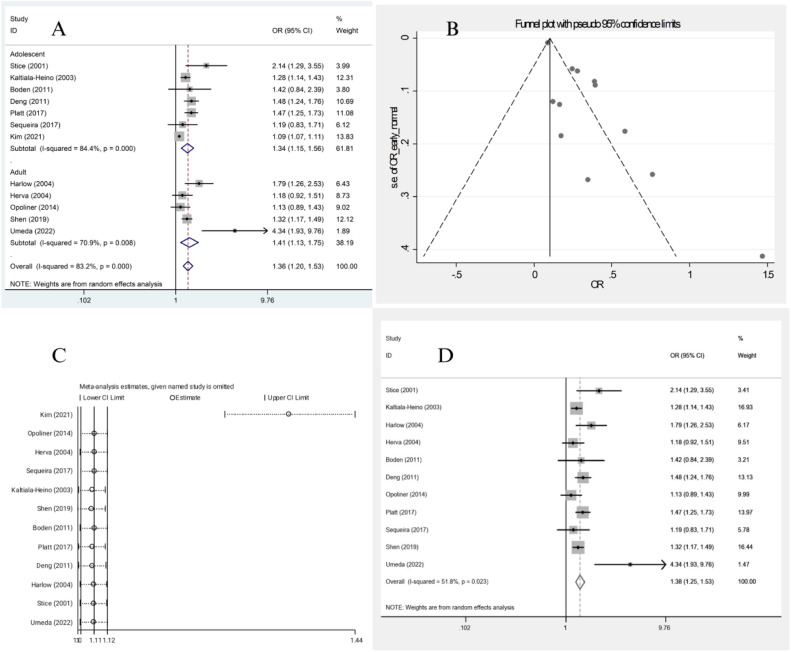
(A) Forest plot diagram of odds of depression among early AAM cases compared to normal AAM controls by included primary studies and 95 % CI, (B) Publication bias assessment with Funnel plot, (C) Sensitivity analysis to assess the impact of each primary study on the odds of depression among early AAM cases compared to normal AAM controls by included primary studies with fixed effect model (showing significant effect by Kim et al. (2021) study), (D) Forest plot diagram of odds of depression among early AAM cases compared to normal AAM controls by included primary studies and 95 % CI excluding Kim et al. (2021) study.

Heterogeneity indices (*I*^2^ = 83.2 %, *Q* = 65.44, *p* < 0.001) indicated high levels of heterogeneity among the results of the primary studies. By combining the results of these twelve studies, the overall odds of depression in females with early menarche were estimated to be significantly higher compared to the normal group (OR = 1.36, 95 % CI 1.20‒1.53) (Fig. 2A). Furthermore, the odds of depression were still significantly higher in females with early menarche compared to the normal AAM group in both adults and adolescents (OR = 1.41, 95 % CI 1.13‒1.75, and OR = 1.34, 95 % CI 1.15‒1.56, respectively).

Subgroup analysis was conducted based on population type, study design, and depression assessment tools which revealed a reduction in heterogeneity when stratified by study design (*I*^2^ from 83.2 % overall to 27.7 % in cohort subgroup), and higher odds of depression among cross-sectional studies (OR = 1.40 in cross-sectional studies vs. OR = 1.26 in cohort studies), and among patients assessed by DSM-IV compared to other tools (Supplementary Fig. 1).

Meta-regression analysis demonstrated that the population type (adults or adolescents) (β = 0.417, *p* = 0.416) (Table 4), study design (β = −0.34, *p* = 0.506), and the diagnostic tool (β = 0.41, *p* = 0.150) do not affect the association between AAM and depression.

**Table 4:** Pooled estimate of the odds ratio of depression by menarche age.

**AAM**	**Number of evidence**	**Sample size**		**Pooled OR (95 % CI) of depression**	**Publication bias**		**Sensitivity analysis (yes/no)**	**Heterogeneity**		**Meta-regression (population)**	
		**Exposed**	**Non-exposed**		**β**	**p-value**		**I-square (%)**	**p-value for *Q***	**β**	**p-value**
Early vs. Normal	12	91,726	326,164	1.36 (1.20‒1.53)	2.28	<0.001	Yes	83.2	<0.001	0.417	0.416
Late vs. Normal	10	14,623	40,833	0.91 (0.76‒1.09)	−0.64	0.718	No	80.6	<0.001	0.179	0.440
Early vs. Late	9	10,389	8464	1.52 (1.22‒1.90)	0.41	0.818	No	79.2	<0.001	−0.248	0.661

The funnel plot (Fig. 2B) and Egger’s test results indicated publication bias (β = 2.28, *t* = 5.16, *p* < 0.001). Thus, the Trim and Fill analysis was conducted and indicated the estimation of two other studies, resulting in the overall odds of 1.30 (95 % CI: 1.15‒1.46, *p* < 0.001).

Sensitivity analysis by leave-one-out method revealed that Kim et al. (2021) study had a significant impact on the overall estimate (Fig. 2C). After omitting this study, the overall odds of depression in females with early menarche were still estimated to be significantly higher compared to the normal group (OR = 1.38, 95 % CI: 1.25‒1.53) (Fig. 2D).

### Late versus normal AAM

Ten studies compared the odds of depression between late and normative AAM (five studies in adolescence,^18^^,^^20^^,^^21^^,^^23^^,^^25^ and five studies in adults[17,19,22,24,26]). These studies included a total of 14,623 participants in the late AAM group and 40,833 individuals in the normal AAM group. In four studies,^17^^,^^18^^,^^24^^,^^25^ the odds of depression were higher among females with late AAM compared to those with a normal AAM; however, only the results of one study were statistically significant.^17^ Also, in six studies, the odds of depression were lower among females with late AAM compared to those with a normal AAM; however, only the results of one study was statistically significant.^21^

**Fig. 3:**
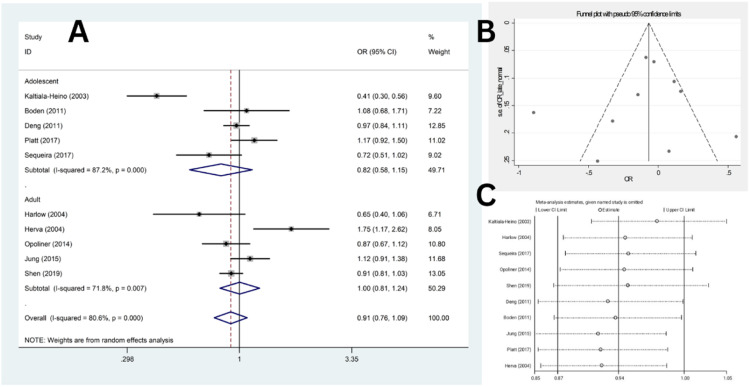
(A) Forest plot diagram of odds of depression among late AAM cases compared to normal AAM controls by included primary studies and 95 % CI; (B) Publication bias assessment with Funnel plot; (C) Sensitivity analysis to assess the impact of each primary study on the odds of depression among late AAM cases compared to normal AAM controls by included primary studies with fixed effect model.

Heterogeneity indices (*I*^2^ = 80.6 %, *Q* = 46.50, *p* < 0.001) indicated high levels of heterogeneity among the results of the primary studies. By combining the results of these ten studies, the overall odds of depression in females with late menarche were not significantly different compared to the normal group (OR = 0.91, 95 % CI: 076‒1.09) (Fig. 3A). Same results were found in adults and adolescents.

Subgroup analysis based on population type, study design, and depression assessment tools revealed no reduction in heterogeneity (Supplementary Fig. 2 and Fig. 3A). Similarly, meta-regression analysis demonstrated no significant results (β = 0.179, *p* = 0.440 for population type, β = 0.24, *p* = 0.290, and β = 0.078, *p* = 0.604 for diagnostic tool). The funnel plot (Fig. 3B) and Egger’s test results indicated no significant publication bias (β = −0.64, *t* = −0.37, *p* = 0.718). Additionally, sensitivity analysis revealed that the effect of each study on the overall estimate was not significant (Fig. 3C).

### Early versus late AAM

Nine studies compared the odds of depression between late and early AAM (five studies in adolescence,^18^^,^^20^^,^^21^^,^^23^^,^^25^ and four studies in adults[17,19,22,26]). These studies included a total of 8464 participants in the late AAM group and 10,389 individuals in the early AAM group. In eight studies, the odds of depression were higher among females with early AAM compared to those with late AAM; however, only the results of five studies were statistically significant. Also, in one study, the odds of depression were lower among females with early AAM compared to those with late AAM, which was not statistically significant.^17^

**Fig. 4:**
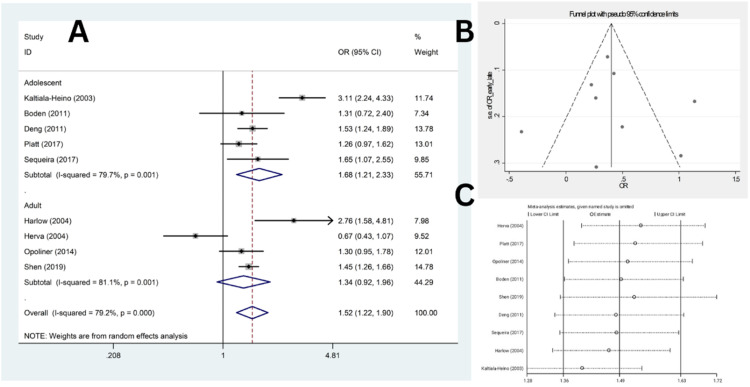
(A) Forest plot diagram of odds of depression among early AAM cases compared to late AAM controls by included primary studies and 95 % CI, (B) Publication bias assessment with Funnel plot, (C) Sensitivity analysis to assess the impact of each primary study on the odds of depression among early AAM cases compared to late AAM controls by included primary studies with fixed effect model.

Heterogeneity indices (*I*^2^ = 79.2 %, *Q* = 38.55, *p* < 0.001) indicated considerable levels of heterogeneity among the results of the primary studies. By combining the results of these studies, the overall odds of depression in females with early menarche were significantly higher compared to the late AAM group (OR = 1.52, 95 % CI: 1.22‒1.90, *p* < 0.001) (Fig. 4A). Furthermore, although the odds of depression was still significantly higher in females with early menarche compared to the normal AAM group among adolescents (OR = 1.68, 95 % CI 1.21‒2.33), the subgroup analysis showed no significant association among adults (OR = 1.34, 95 % CI: 0.92‒1.96).

Subgroup analysis was conducted based on study design and depression assessment tools which revealed a reduction in heterogeneity when stratified by study design (*I*^2^ from 79.2 % overall to 64.6 % in cohort subgroup), and higher odds of depression among cross-sectional studies (OR = 1.79 in cross-sectional studies vs. OR = 1.18 in cohort studies), and among patients assessed by CES-D compared to other tools (Supplementary Fig. 3). Meta-regression analysis demonstrated no significant results (β = −0.248, *p* = 0.661 for population type, β = −0.75, *p* = 0.135 for study design, and β = −0.14, *p* = 0.677 for diagnostic tool). The funnel plot (Fig. 4B) and Egger’s test results indicated no significant publication bias (β = 0.41, *t* = 1.75, *p* = 0.818). Additionally, sensitivity analysis revealed that the effect of each study on the overall estimate was not significantly different (Fig. 4C).

## Discussion

The aim of this systematic review and meta-analysis was to evaluate current evidence to clarify the association between AAM and depression. Thirteen primary studies were included, and comparisons were made between early, late, and normal AAM regarding the odds of depression. The present results demonstrated that the odds of depression are 30 % higher in females with early menarche compared to normal menarche (after trim and fill analysis) and 52 % higher in females with early menarche compared to late menarche. Also, late AAM has shown decreased odds of depression compared to normative AAM; however, this association was not statistically significant. The association between AAM and depression might present with a dose-responsive behavior since with later ages at menarche, the odds of depression decrease (OR = 1.52 vs. 1.30 vs.0.91).

This meta-analysis indicated a significant link between early menarche and an elevated odd of depression, with a possible dose-response relationship between AAM and the likelihood of depression. Several factors may explain these findings. Early menarche results in earlier exposure to estrogen and other sex hormones, which are known to play a role in mood regulation, and earlier exposure to fluctuations in ovarian hormones.^29^^,^^30^

The premature onset of these hormonal changes may heighten the risk of depression, particularly during adolescence.^31^ Estrogen influences brain function through modulation of emotional perception, mood regulation, and stress responses which are pivotal factors in the development of depression. Additionally, estrogen interacts with various neurotransmitter systems, such as serotonergic, dopaminergic, and acetylcholine which are crucial for mood regulation, nervous irritability, behavior, and cognitive function.^32^

Estrogen enhances dopaminergic signaling in mesolimbic pathways and supports cholinergic function, which is critical for attention and memory. Disruptions in these systems due to early hormonal exposure may contribute to cognitive biases and emotional dysregulation seen in depression.^30^^,^^33^^,^^34^ Estrogen also modulates inflammatory pathways, with low estrogen states associated with elevated pro-inflammatory cytokines (e.g., IL-6, TNF-α), which are implicated in depression. Early menarche may accelerate immune system maturation, potentially increasing lifelong vulnerability to inflammation-related mood disorders.^30^^,^^35^^,^^36^

The timing of puberty can significantly influence neurodevelopmental processes, particularly in brain regions related to emotional regulation, such as the prefrontal cortex and the amygdala.^37^ The hippocampus and amygdala, key regions for emotional regulation, are rich in Estrogen Receptors (ERα and ERβ). Preclinical studies show that estrogen administration reduces anxiety- and depressive-like behaviors in rodents by promoting dendritic spine formation and synaptic plasticity in these regions. However, *timing* is critical, as early or aberrant estrogen surges may disrupt normative synaptic pruning and connectivity, exacerbating stress sensitivity.^38^^,^^39^

Early menarche may alter the development of these areas, potentially increasing susceptibility to mood disorders like depression.^40^ Additionally, early puberty can lead to changes in the Hypothalamic-Pituitary-Adrenal (HPA) axis, the body's central stress response system.^37^ Dysregulation of the HPA axis is associated with both early puberty and depression, suggesting a possible shared biological pathway.^40^

Early menarche is associated with heightened HPA axis reactivity, a feature common in depression. Estrogen potentiates stress-induced cortisol release, and premature activation of this axis may lead to maladaptive stress responses. Animal models demonstrate that estrogen’s effects on anxiety and depression are dose- and duration-dependent, with physiological levels being protective but supraphysiological or mistimed exposure exacerbating dysregulation.^39^^,^^41^^,^^42^

Emerging evidence highlights ERβ (a subtype of estrogen receptors) as particularly relevant to mood. ERβ knockout mice exhibit increased anxiety and depressive behaviors, and selective ERβ agonists have antidepressant effects.^39^^,^^43^, ^44^, ^45^ Early menarche might perturb the balance between ERα and ERβ signaling, contributing to emotional dysregulation.

Beyond estrogen, the interplay of cortisol, progesterone, and other hormones during puberty may further amplify depression risk. Early puberty coincides with increased cortisol secretion in response to psychosocial stressors leading to higher depressive symptoms.^46^^,^^47^ Progesterone, which fluctuates cyclically with estrogen, modulates mood through its metabolites such as Allopregnanolone. Allopregnanolone enhances GABA receptor activity, promoting inhibitory tone and stress resilience. Lower allopregnanolone levels are linked to depression and MDD, particularly in women with rapid progesterone withdrawal. Early menarche may disrupt the typical estrogen-progesterone ratio, leading to exaggerated mood swings.^48^^,^^49^

Girls who experience early menarche often undergo accelerated physical development compared to their peers, which can lead to body image concerns and self-esteem issues, both of which are established risk factors for depression.^4^^,^^50^^,^^51^ Nonetheless, early maturation could lead to self-harming behaviors, earlier pregnancies, and substance abuse which increases the risk of depression.^52^^,^^53^ Additionally, the social impact of appearing different from peers can lead to feelings of isolation or social anxiety, further increasing the risk of depression.^54^

On the other hand, research has also demonstrated that earlier menarche could be the consequence of being raised in stressful circumstances, especially in the family such as high levels of family conflict, divorce, father's absence, or sexual abuse, which increase the odds of depression.^55^, ^56^, ^57^ Thus, it is still uncertain whether early AAM leads to depression or vice versa, and whether depression and early menarche are both consequences of another set of variables. The authors suggest that future research be designed in a way that addresses these questions.

Furthermore, the interpretation of these findings may be influenced by the variability in depression assessment scales used across studies. While this meta-analysis pooled data across studies using validated instruments, the diversity in tools (such as BDI, PHQ-9, etc.) introduces potential variability in the assessment of depressive symptoms. Each scale has unique thresholds, focuses on different dimensions of depressive symptoms, and varies in sensitivity and specificity, which may impact how depression is diagnosed and interpreted.

This heterogeneity in measurement could complicate comparisons across studies and challenge the robustness of the pooled conclusions. However, meta-regression and subgroup analyses analysis did not show a significant effect on this matter. Nevertheless, exploring the influence of these scales further highlights the importance of standardizing depression assessments in future research to improve data consistency and interpretability. Additionally, future studies should evaluate whether certain depression scales are more or less likely to detect specific patterns of association between AAM and depression, which may provide a more nuanced understanding of these relationships.

The dose-response relationship between AAM and depression risk may reflect the cumulative impact of various risk factors. Girls who experience early menarche may encounter more stressors and negative experiences over time compared to those with later menarche, resulting in a higher overall risk of depression. The elevated risk of depression associated with earlier menarche could be due to prolonged exposure to hormonal influences and social stressors. In contrast, girls who experience menarche later might face a shorter period of vulnerability, reducing the impact of these combined factors.

Early maturers would experience less preparation time to develop the skillsets and resources required to face physical maturation compared to later maturing peers. Thus, this may lead to higher levels of anxiety and depression.^58^^,^^59^ Additionally, in some cultures, the idea of menstruation and menstrual discharge is regarded as unsanitary, thus, females who are not in their menstruation period are regarded as sanitary and healthy adults while females who are menstruating are thought to be unhealthy, and insanitary resulting in higher levels of anxiety and depression.^32^

### Limitations and future research directions

Nonetheless, this review is limited by the nature of the studies included. Cross-sectional studies are inherently unsuitable for establishing cause-effect relationships. Although the authors found associations between AAM and depression, the temporal direction of this relationship remains unclear, as cross-sectional design cannot determine whether early menarche increases the risk of depression or vice versa.

However, the authors would like to highlight that for certain independent variables like genetic factors, blood type, and AAM, we can be reasonably confident about the temporal precedence of exposure, even in cross-sectional studies. As demonstrated by the subgroup analysis, the odds of depression were higher among cross-sectional studies which could indicate the over-exaggeration bias within this type of study design. Also, the heterogeneity of studies was lower among cohorts compared to cross-sectional studies indicating study design is a potential source of heterogeneity. Future research should prioritize longitudinal studies to better clarify the causal pathways underlying these associations.

The observed publication bias suggests that smaller studies reporting non-significant or null associations between early menarche and depression may have been less likely to be published. This could lead to an overestimation of the pooled effect size in the present meta-analysis. For instance, the initial OR of 1.36 for early versus normative AAM was attenuated to 1.30 after the Trim and Fill adjustment, indicating modest inflation of the original estimate.

While Trim and Fill is a widely used tool to correct for publication bias, it operates under the assumption that the bias stems primarily from small-study effects (e.g., selective publication of statistically significant results). However, this method cannot account for other sources of bias. While publication bias remains a limitation, the consistency of these findings across sensitivity analyses and the persistence of a statistically significant association after Trim and Fill adjustment support the robustness of the primary conclusion.

The potential for selection bias is another notable limitation. Most of the studies were conducted in high-income countries, with populations from North America, Europe, and parts of Asia. Regions such as Africa, the Middle East, and Latin America, where cultural and environmental factors may influence both AAM and depression risk, were underrepresented. Expanding research to include more diverse geographic regions and cultural contexts is critical to ensuring the broader applicability of these findings.

Another key limitation is the insufficient consideration of confounding factors that could influence both AAM and depression due to the lack of sufficient data in the primary studies. While some included studies adjusted for covariates, variables such as Body Mass Index (BMI), family history of depression, early life adversity, and socioeconomic status were not consistently accounted for. The authors recommend future studies rigorously control for these factors to isolate the independent effect of AAM.

### Clinical implications

The findings of this meta-analysis underscore the importance of recognizing early menarche as a potential risk marker for depression. The robust association between early menarche and elevated odds of depression highlights several clinical opportunities for early identification, intervention, and prevention:

• Screening and early detection

Healthcare providers, particularly pediatricians, family physicians, and school health services, should consider incorporating AAM into routine mental health screenings. Early menarche could serve as a *red flag* prompting closer monitoring for depressive symptoms, especially in high-risk populations (e.g., those with family history of depression or adverse childhood experiences). Standardized tools (e.g., PHQ-9 for adolescents) could be administered during well-visits for girls with early menarche to facilitate early diagnosis.

• Targeted interventions

Girls with early menarche and their families may benefit from Mindfulness-based stress reduction, cognitive-behavioral therapy, and counseling about the potential psychological impacts of early puberty, including body image concerns and social challenges. Education on coping strategies and stress management could mitigate risk. Schools could implement peer support groups or resilience-building programs for early-maturing girls to address feelings of isolation and social stigma.

• Preventive strategies

Parents of early-maturing girls should be educated about signs of depression and encouraged to foster open communication about emotional well-being. Public health campaigns could reduce stigma around early puberty and promote mental health literacy in communities where cultural attitudes may exacerbate distress (e.g., menstrual taboos).

• Research and policy directions

Longitudinal studies are needed to clarify causality and explore bidirectional relationships (e.g., whether childhood adversity accelerates menarche and increases depression risk). Clinical guidelines could consider incorporating AAM as part of depression risk stratification tools, particularly in adolescent health settings.

## Conclusion

The present findings indicate that early menarche is associated with elevated odds of depression compared to females with both normative and late AAM. While these results highlight a potential relationship between the timing of menarche and mental health outcomes, the predominance of cross-sectional studies among the included research limits the ability to draw definitive conclusions about causality. Thus, future research should prioritize longitudinal designs to better understand the temporal and potentially bidirectional nature of the association between AAM and depression.

## Availability of data and materials

All data generated or analyzed during this study are included in this article.

## Ethics approval and consent to participate

Not applicable.

## Consent for publication

Not applicable.

## Funding

None.

## CRediT authorship contribution statement

**Erfan Ghadirzadeh:** Conceptualization, Data curation, Project administration, Investigation, Software, Visualization, Writing – original draft, Writing – review & editing. **Mahmood Moosazadeh:** Conceptualization, Formal analysis, Methodology, Project administration, Software, Validation, Visualization, Writing – review & editing. **Kiarash Shakeriastani:** Data curation, Project administration, Writing – review & editing. **Maryam Zarrinkamar:** Data curation, Investigation, Writing – review & editing. **Mobina Gheibi:** Conceptualization, Data curation, Resources, Investigation, Visualization, Writing – review & editing. **Forouzan Elyasi:** Resources, Investigation, Supervision, Validation, Writing – review & editing. **Mojgan Geran:** Investigation, Writing – review & editing.

## Declaration of competing interest

The authors declare that they have no known competing financial interests or personal relationships that could have appeared to influence the work reported in this paper.

## Appendix Supplementary materials

Image, application 1

Image, application 2

Image, application 3
