# Supplementary material for: The association between age at menarche and depression: A systematic review and meta-analysis with meta-regression
Source: Clinics (Sao Paulo). 2025 May 23;80:100695. doi: 10.1016/j.clinsp.2025.100695 (PMC12148817; doi:10.1016/j.clinsp.2025.100695)

**CLINICS-D-24-01185_Supplementary Materials**

**Supplementary Figure 1** (A) Subgroup analysis forest plot diagram of odds of depression among early AAM cases compared to normal AAM controls by included primary studies and 95% CI based on study design, (B) Subgroup analysis forest plot diagram of odds of depression among early AAM cases compared to normal AAM controls by included primary studies and 95% CI based on depression assessment tools.


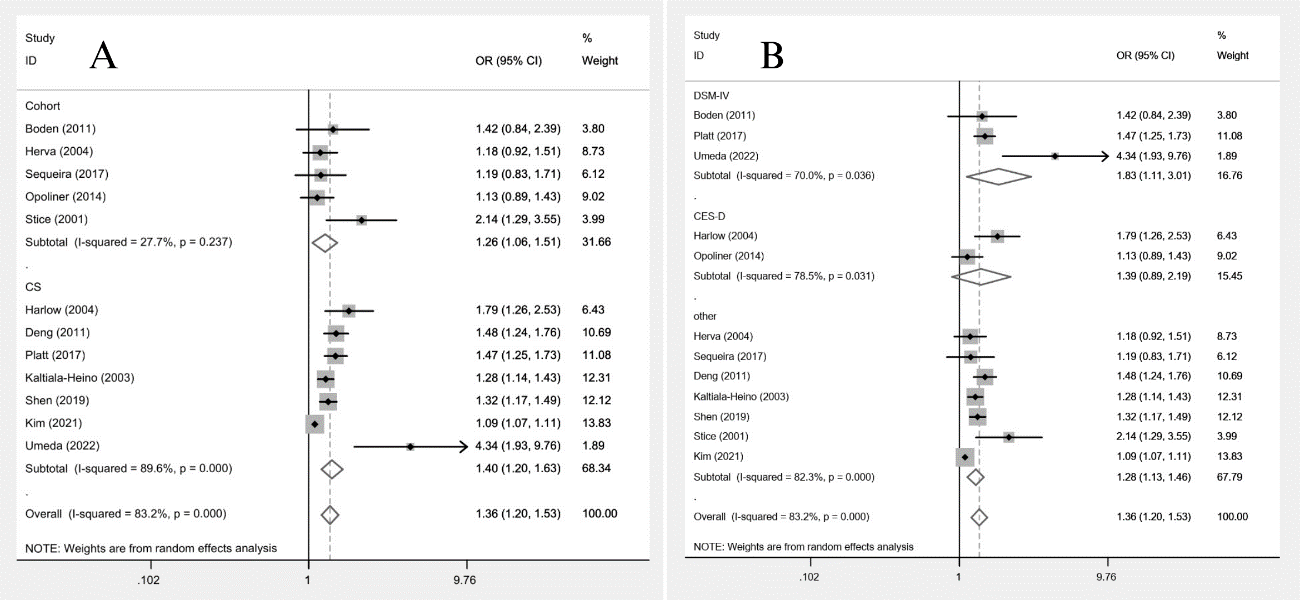


**Supplementary Figure 2** (A) Subgroup analysis forest plot diagram of odds of depression among late AAM cases compared to normal AAM controls by included primary studies and 95% CI based on study design, (B) Subgroup analysis forest plot diagram of odds of depression among late AAM cases compared to normal AAM controls by included primary studies and 95% CI based on depression assessment tools.


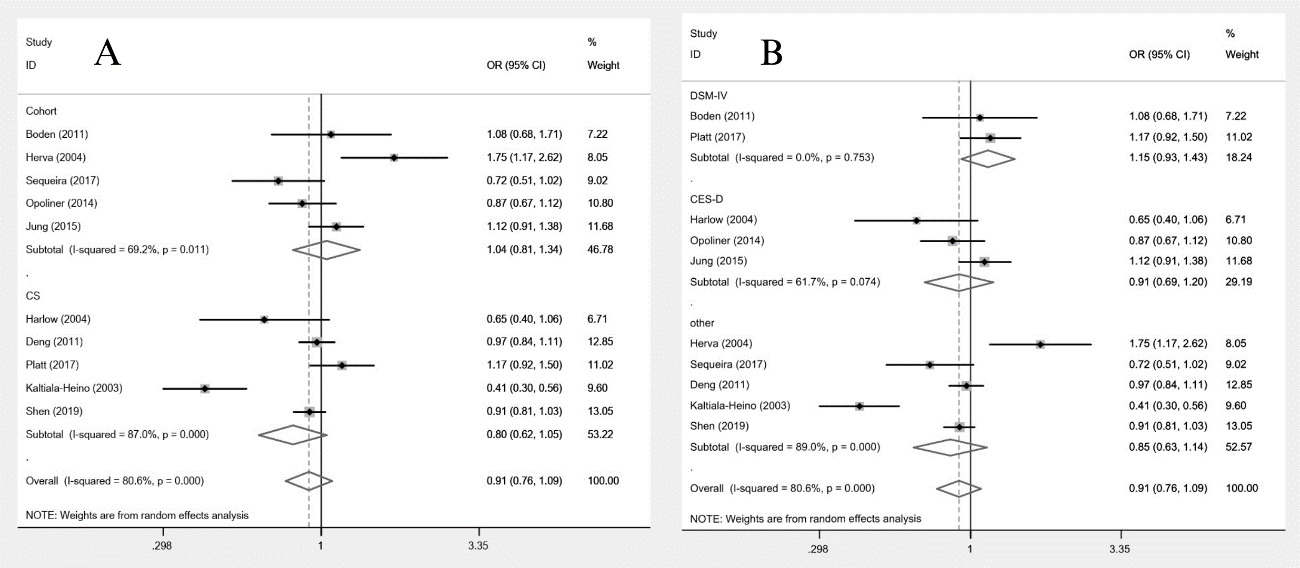


**Supplementary Figure 3** (A) Subgroup analysis forest plot diagram of odds of depression among early AAM cases compared to late AAM controls by included primary studies and 95% CI based on study design, (B) Subgroup analysis forest plot diagram of odds of depression among early AAM cases compared to late AAM controls by included primary studies and 95% CI based on depression assessment tools.


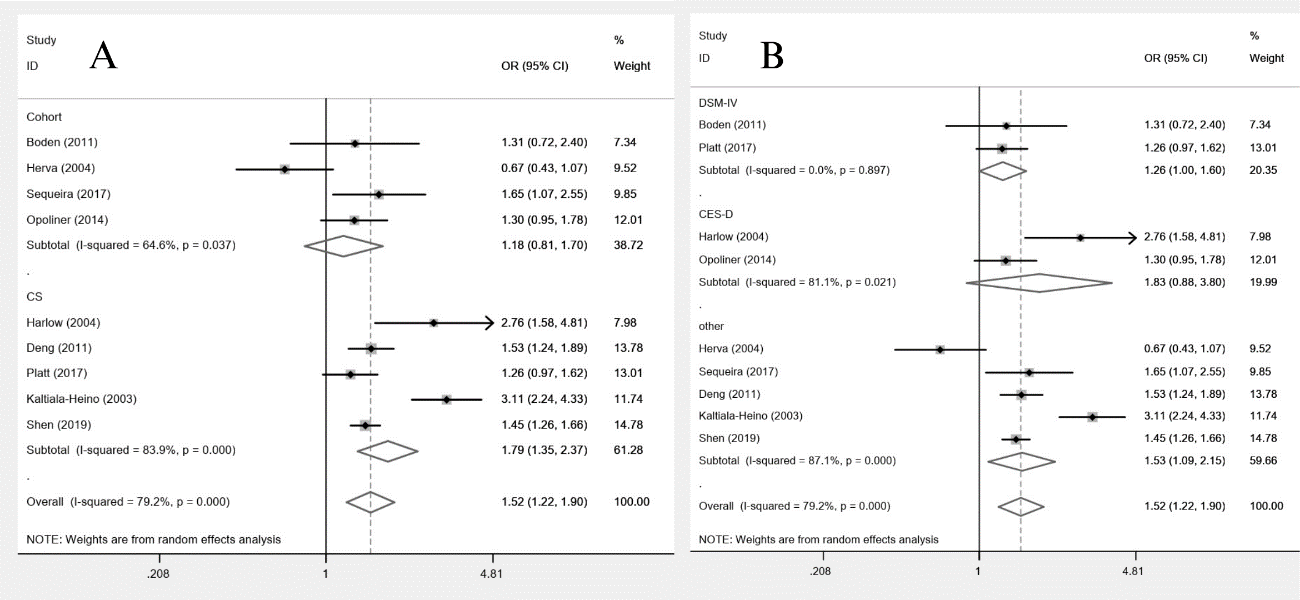

Supplement: Supplementary file 1 [file mmc1.docx]
