# Supplementary material for: The association between age at menarche and depression: A systematic review and meta-analysis with meta-regression
Source: Clinics (Sao Paulo). 2025 May 23;80:100695. doi: 10.1016/j.clinsp.2025.100695 (PMC12148817; doi:10.1016/j.clinsp.2025.100695)
Supplement: Supplementary file 3 [file mmc3.docx]

**PubMed**

("Age at menarche"[tiab] OR "Menarche"[tiab] OR "menarche age"[tiab] OR "menarchal age"[tiab] OR "Early menarche"[tiab] OR "Late menarche"[tiab] OR "First menstrual cycle"[tiab] OR "Mensturation"[tiab] OR "Menstural"[tiab]) AND ("Depression"[tiab] OR "Depressive"[tiab])

**Embase**

(Menarche:ti,ab OR Mensturation:ti,ab OR Menstural:ti,ab OR ‘menarche age’:ti,ab OR ‘menarchal age’:ti,ab) AND (Depression:ti,ab OR Depressive:ti,ab)

**Scopus**

(TITLE-ABS-KEY("Age at menarche") OR TITLE-ABS-KEY("Menarche") OR TITLE-ABS-KEY("Early menarche") OR TITLE-ABS-KEY("Late menarche") OR TITLE-ABS-KEY("First menstrual cycle") OR TITLE-ABS-KEY("Mensturation") OR TITLE-ABS-KEY("Menstural") OR TITLE-ABS-KEY("menarche age") OR TITLE-ABS-KEY("menarchal age")) AND (TITLE-ABS-KEY("Depression") OR TITLE-ABS-KEY("Depressive"))

**Web of Science**

ALL=((Age at menarche) OR (Menarche) OR (Menarche age) OR (Menarchal age) OR (Early menarche) OR (Late menarche) OR (First menstrual cycle) OR (Mensturation) OR (Menstural))

AND

ALL=((Depression) OR (Depressive))
